# Synthesis and crystal structure of *trans*-di­chlorido[3-methyl-1-(4-vinyl­benz­yl)-1*H*-imidazol-3-ium-2-yl-κ*C* ^2^](4-phenyl­pyridine-κ*N*)palladium(II)

**DOI:** 10.1107/S2056989016004394

**Published:** 2016-03-22

**Authors:** Maitham H. Majeed, Ola F. Wendt

**Affiliations:** a Centre for Analysis and Synthesis, Department of Chemistry, Lund University, Box 124 221 00, Lund, Sweden

**Keywords:** crystal structure, palladium, N-heterocyclic carbenes, monomers for polymerization, 1-methyl-3-(4-vinyl­benz­yl)imidazole

## Abstract

The title compound, [PdCl_2_(C_11_H_9_N)(C_13_H_14_N_2_)], represents a new class of palladium-based polymerizable monomer which could give a potentially catalytically active polymer. It was synthesized *via* transmetallation from the corresponding silver complex. The Pd^II^ ion coordinates two Cl anions, one C atom from the N-heterocyclic carbene (NHC) ligand and one N atom from the 4-phenyl­pyridine ligand, displaying a slightly distorted square-planar geometry. The dihedral angle between the imidazole ring and the pyridine ring is 34.53 (8)°. The Pd—C bond length between the NHC ligand and the Pd^II^ ion is 1.9532 (16) Å. In the crystal, weak non-classical C—H⋯Cl hydrogen bonds link the mol­ecules into a tape structure along [101]. A weak π–π inter­action is also observed [centroid–centroid distance = 3.9117 (11) Å].

## Abstract 2

In the title compound, [Pd(C_11_H_9_N)(C_13_H_14_N_2_)Cl_2_], the Pd^II^ ion is coordinated by two Cl anions, one carbene C atom and one pyridine N atom in a slightly distorted square-planar geometry. In the crystal, the mol­ecules are linked through weak C—H⋯Cl hydrogen bonds into a tape structure.

## Chemical context   

In the last few years, palladium complexes with N-heterocyclic carbene ligands (Pd-NHCs) have received attention, *inter alia* as catalysts for cross-coupling in organic synthesis (Hadei *et al.*, 2005 ▸; Nasielski *et al.*, 2010 ▸; Valente *et al.*, 2010 ▸, 2012 ▸). NHC complexes derived from vinyl imidazolium salts are of growing significance in organometallic transformations because of their potential as precursors in heterogeneous catalysis, biocompatibility, anti-microbial activity and fuel cell applications (Dani *et al.*, 2015 ▸; Ghazali-Esfahani *et al.*, 2013 ▸; Anderson & Long, 2010 ▸; Kim *et al.*, 2005 ▸; Kuzmicz *et al.*, 2014 ▸; Seo & Chung, 2014 ▸; Li *et al.*, 2011 ▸). The crystal structures of 1-methyl-3-(4-vinyl­benz­yl) imidazolium hexa­fluorido­phosphate and silver complexes with 1-methyl-3-(4-vinyl­benz­yl) imidazole as a carbene ligand have been reported previously (Lu *et al.*, 2009 ▸, 2010 ▸). Here we report on the crystal structure of a new type of Pd-NHC complex belonging to the group of PEPPSI (pyridine-enhanced precatalyst preparation stabilization and initiation) catalysts, which are stable towards air and moisture, and have the advantage of being easy to synthesize and handle (Hadei *et al.*, 2005 ▸).

## Structural commentary   

**Figure 1:**
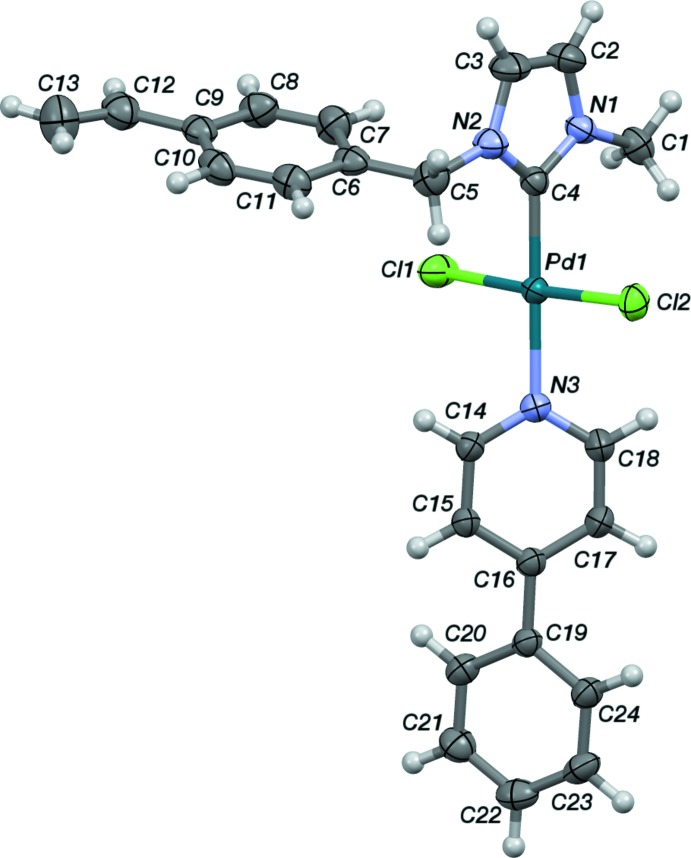
The mol­ecular structure of the title compound (**4**). All non-H atoms are represented as displacement ellipsoids drawn at the 50% probability level and H atoms as small spheres with arbitrary radii.

In the title compound, the Pd^II^ ion coordinates the five-membered NHC ligand with a Pd1—C4 bond length of 1.9532 (16) Å and the 4-phenyl­pyridine ligand with a Pd1—N3 bond length of 2.0938 (14) Å. The two mutually *trans* Cl ions fulfil the coordination sphere (Fig. 1 ▸). Bond angles in the so-formed distorted square-plane are all close to 90° with the C4—Pd1—Cl angles slightly less than 90° and the others slightly more. The C4—Pd1—N3 angle shows an expected value 179.52 (6)°, while Cl1—Pd1—Cl2 exhibits a slightly distorted angle of 176.789 (17)°, probably due to the steric influence of the aromatic rings (Sevinçek *et al.*, 2007 ▸). The dihedral angle between the N1/C4/N2/C3/C2 and C6–C11 rings in the NHC ligand is 77.90 (5)°.
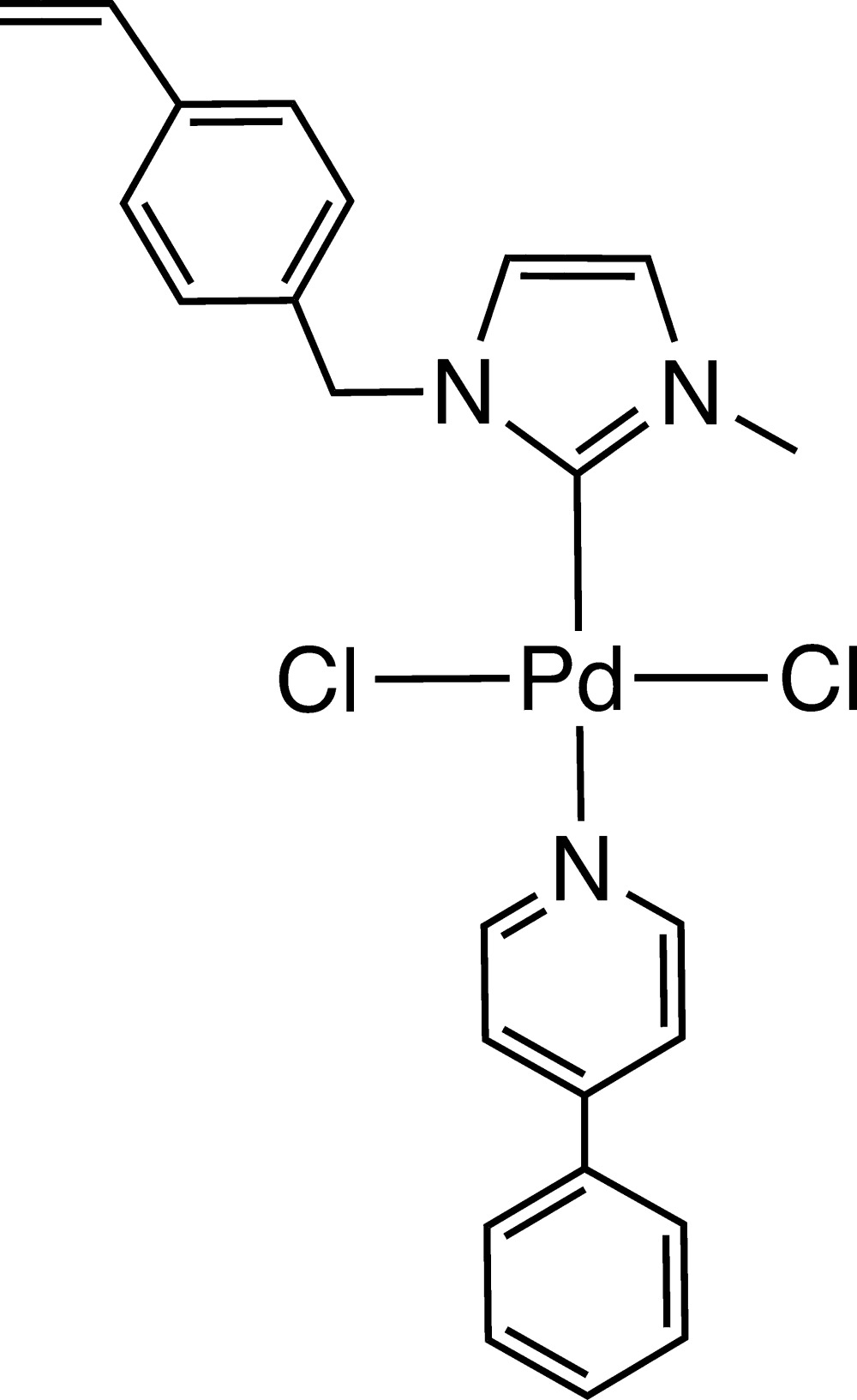


 The dihedral angles between the N1/C4/N2/C3/C2 ring on one hand and the N3/C14–C18 and C19–C24 rings on the other are 34.53 (8) and 65.78 (7)°, respectively. The C12—C13 bond length of the vinyl group is 1.299 (3) Å, corroborating the double-bond character. The same goes for the C2—C3 distance which is 1.330 (3) Å. The N2—C4—Pd1—N3, N1—C4—Pd1—Cl2, C18—N3—Pd1—Cl2 and C17—C16—C19—C24 torsion angles are −30 (7), 81.15 (15), −49.40 (15) and 32.42 (3)°, respectively. A Cambridge Structural Database (CSD) search to validate the Pd—Cl and Pd—N bonding was performed over 47 entries. The Cl—Pd—Cl and N—C—N angles range from 170 to 180° and from 104.8 to 106.2°, respectively; the Pd—Cl bond lengths are in the range 2.286–2.318 Å. The bond lengths and angles of the title compound **4** are comparable to the literature values.

## Supra­molecular features   

**Figure 2:**
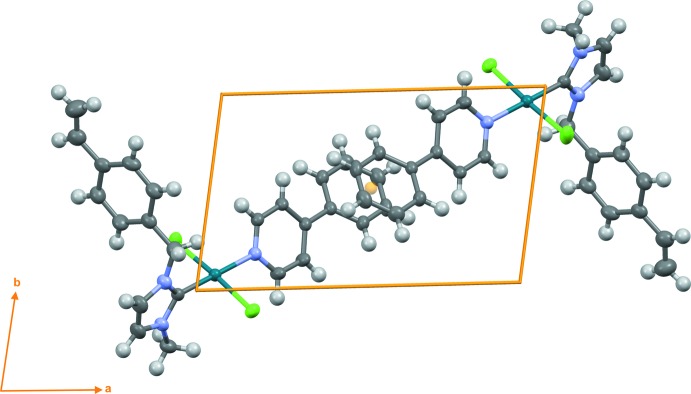
The dimer of the title compound (**4**) linked through the π–π inter­action.

**Figure 3:**
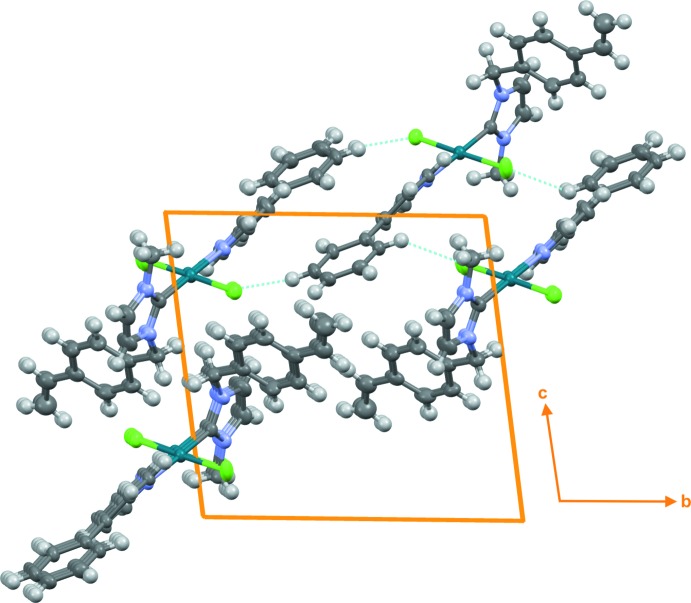
A crystal packing diagram of the title compound (**4**). The non-classical C—H⋯Cl hydrogen bonds are shown by dotted lines.

In addition to dispersion inter­actions, the crystal of title compound **4** shows a π–π inter­action between the C19–C24 phenyl rings of neighbouring mol­ecules with a centroid–centroid distance of 3.9117 (11) Å (Fig. 2 ▸). Two weak non-classical C—H⋯Cl hydrogen bonds are detected (Table 1 ▸). No C—H⋯π contacts are present in the crystal packing diagram of compound **4** (Fig. 3 ▸).

## Synthesis and crystallization   

**Figure 4:**
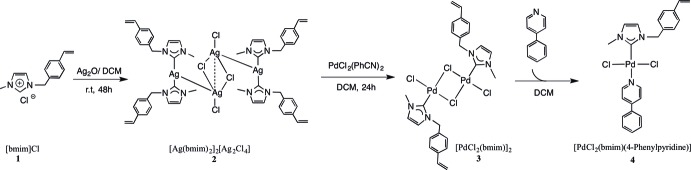
Synthesis pathway of the title compound (**4**).

***General:*** Solvents and chemicals were purchased from commercial suppliers and used as received. The imidazolium salts **1** and **2** were prepared according to previously reported procedures (Kim *et al.*, 2005 ▸; Lu *et al.*, 2009 ▸). The title compound **4** was synthesized according to the carbene silver(I) route, as shown in Fig. 4 ▸. Transmetallation of the ligand from the tetra­meric silver complex **2** gave the chlorido-bridged palladium dimer **3**. Cleavage of the dimer with phenyl­pyridine afforded complex **4** in excellent yield. With its vinyl groups it can serve as a precursor in co-polymerization reactions with *e.g.* styrene to form polymeric materials with catalytic properties.

**[PdCl_2_(bmim)]_2_** (**3**). A 100 ml Schlenk flask was charged with **2** (7.0 g, 20.5 mmol), 50 ml of dry CH_2_Cl_2_ and Pd(PhCN)_2_Cl_2_ (7.8 g, 20.5 mmol). The mixture was stirred for 48 h at room temperature, during which time the solution changed colour to cloudy light brown. It was filtered through Celite and the filtrate was reduced to *ca* 10 ml. Upon addition of *n*-hexane, a light-brown solid was formed, which was collected on a frit and dried under vacuum to give 5.97 g (yield 78%).

**[PdCl_2_(bmbim)(4-Phenyl­pyridine)]** (**4**). 4-Phenyl­pyridine (0.085 g, 0.55 mmol) was added to a 40 ml solution of **3** (0.25 g, 0.26 mmol) in dry CH_3_CN and stirred at ambient temperature for 24 h, during which time the solution changed colour to clear yellow. The mixture was filtered through Celite and all solvents were evaporated. The solids were dissolved in CH_2_Cl_2_ and, upon addition of *n*-hexane, a yellow solid was formed, which was collected on a frit and dried under vacuum to give 0.153 g (93%) of **4**.

Single crystals of **4** suitable for X-ray diffraction were obtained by slow diffusion of *n*-hexane into a saturated CH_2_Cl_2_ solution of the compound.

## Refinement details   

Crystal data and structure refinement details are summarized in Table 2 ▸. H atoms were treated as riding, with C—H = 0.95–0.99 Å, and with *U*_iso_(H) = 1.2*U*_eq_(C).

## Supplementary Material

Crystal structure: contains datablock(s) Global, I. DOI: 10.1107/S2056989016004394/is5447sup1.cif

Structure factors: contains datablock(s) I. DOI: 10.1107/S2056989016004394/is5447Isup2.hkl

Numbering for the assignment of NMR spectra. DOI: 10.1107/S2056989016004394/is5447sup3.tif

CCDC reference: 1468135

Additional supporting information:  crystallographic information; 3D view; checkCIF report

## Figures and Tables

**Table 1:** Hydrogen-bond geometry (Å, °)

*D*—H⋯*A*	*D*—H	H⋯*A*	*D*⋯*A*	*D*—H⋯*A*
C20—H20⋯Cl1^i^	0.95	2.81	3.6021 (18)	142
C23—H23⋯Cl2^ii^	0.95	2.74	3.6537 (19)	162

Symmetry codes: (i) 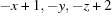; (ii) 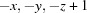.

**Table 2:** Experimental details

Crystal data	
Chemical formula	[PdCl_2_(C_11_H_9_N)(C_13_H_14_N_2_)]
*M*_r_	530.75
Crystal system, space group	Triclinic, *P* 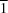
Temperature (K)	183
*a*, *b*, *c* (Å)	7.8768 (3), 12.2939 (5), 12.6120 (4)
α, β, γ (°)	95.692 (3), 97.267 (3), 103.574 (3)
*V* (Å^3^)	1167.09 (8)
*Z*	2
Radiation type	Mo *K*α
μ (mm^−1^)	1.04
Crystal size (mm)	0.39 × 0.27 × 0.1

Data collection	
Diffractometer	Agilent Xcalibur Ruby
Absorption correction	Analytical (*CrysAlis PRO*; Agilent, 2012 ▸)
*T*_min_, *T*_max_	0.727, 0.916
No. of measured, independent and observed [*I* > 2σ(*I*)] reflections	28730, 7116, 6179
*R*_int_	0.037
(sin θ/λ)_max_ (Å^−1^)	0.714

Refinement	
*R*[*F* ^2^ > 2σ(*F* ^2^)], *wR*(*F* ^2^), *S*	0.027, 0.068, 1.04
No. of reflections	7116
No. of parameters	272
H-atom treatment	H-atom parameters constrained
Δρ_max_, Δρ_min_ (e Å^−3^)	0.45, −0.42

Computer programs: *CrysAlis PRO* (Agilent, 2012 ▸), *SUPERFLIP* (Palatinus & Chapuis, 2007 ▸), *SHELXL2013* (Sheldrick, 2015 ▸) and *OLEX2* (Dolomanov *et al.*, 2009 ▸).

## supplementary crystallographic information

### Crystal data

**Table:**

[PdCl_2_(C_11_H_9_N)(C_13_H_14_N_2_)]	*Z* = 2
*M**_r_* = 530.75	*F*(000) = 536
Triclinic, *P*1	*D*_x_ = 1.510 Mg m^−^^3^
*a* = 7.8768 (3) Å	Mo *K*α radiation, λ = 0.71073 Å
*b* = 12.2939 (5) Å	Cell parameters from 11991 reflections
*c* = 12.6120 (4) Å	θ = 2.5–32.8°
α = 95.692 (3)°	µ = 1.04 mm^−^^1^
β = 97.267 (3)°	*T* = 183 K
γ = 103.574 (3)°	Plate, clear light yellow
*V* = 1167.09 (8) Å^3^	0.39 × 0.27 × 0.1 mm

### Data collection

**Table:**

Agilent Xcalibur Ruby diffractometer	7116 independent reflections
Radiation source: Enhance (Mo) X-ray Source	6179 reflections with *I* > 2σ(*I*)
Graphite monochromator	*R*_int_ = 0.037
Detector resolution: 10.4498 pixels mm^-1^	θ_max_ = 30.5°, θ_min_ = 2.5°
ω scans	*h* = −11→11
Absorption correction: analytical (*CrysAlis PRO*; Agilent, 2012)	*k* = −17→17
*T*_min_ = 0.727, *T*_max_ = 0.916	*l* = −18→18
28730 measured reflections	

### Refinement

**Table:**

Refinement on *F*^2^	Primary atom site location: iterative
Least-squares matrix: full	Hydrogen site location: inferred from neighbouring sites
*R*[*F*^2^ > 2σ(*F*^2^)] = 0.027	H-atom parameters constrained
*wR*(*F*^2^) = 0.068	*w* = 1/[σ^2^(*F*_o_^2^) + (0.0295*P*)^2^ + 0.2776*P*] where *P* = (*F*_o_^2^ + 2*F*_c_^2^)/3
*S* = 1.04	(Δ/σ)_max_ = 0.001
7116 reflections	Δρ_max_ = 0.45 e Å^−^^3^
272 parameters	Δρ_min_ = −0.42 e Å^−^^3^
0 restraints	

### Special details

**Table:**

Experimental. NMR spectra were acquired on a Bruker Avance 400 F T–NMR spectrometer (^1^H: 400.1 MHz). Residual solvent peaks were used as an internal reference. Elemental analyses were performed by H. Kolbe Microanalytisches Laboratorium, Mülheim an der Ruhr, Germany. The atomic numbering refers to Figure S1.(3):^1^H NMR (400 MHz, CDCl_3_): δ 7.42 (s, 8H, H-4, H5, H7 and H8), 6.87 (d, *J* = 1.9 Hz, 2H, H11), 6.75–6.65 (m, overlapping, 2H, H2), 6.70 (d, *J* = 1.8 Hz, 2H, H10), 5.82 (s, 4H, H9), 5.76 (d, *J* = 17.6 Hz, 2H, H1_B_), (5.28 (d, *J* = 10.9 Hz, 2H, H1_A_), 4.21 (s, 6H, H12). ^13^C NMR (400 MHz, CDCl_3_): δ 141.7 (C13), 138.2 (C6), 136.3 (C2), 134.2 (C3), 129.3 (C4 and C8), 127.0 (C5 and C7), 121.9 and 124.0 (C10 and C11 of imidazolyl), 114.9 (C1), 54.6 (C9), 38.4 (C12). Anal. Calcd for C_26_H_28_C_l4_N_4_Pd_2_: C, 41.57; H, 3.76; N, 7.46. Found: C, 41.93; H, 4.21; N, 7.22.(4):^1^H NMR (400 MHz, CDCl_3_): δ 9.02 (dd, *J*=5.2, 1.5 Hz, 2H, H14 and H18), 7.65 – 7.4 (m, 9H, H4, H5, H7, H8, H19, H20, H21, H22 and H23), 7.56 (dd, *J*=5.2, *J*=1.6 Hz, H15 and H17), 6.89 (d, *J*=2.0 Hz, 1H, H11), 6.75 – 6.65 (m, overlapping, 1H, H2), 6.72 (d, *J* = 1.8 Hz, 1H, H10), 5.84 (s, 2H, H9), 5.76 (d, *J*=17.6 Hz, 1H, H1_B_), 5.27 (d, *J*=10.9 Hz, 1H, H1_A_), 4.21 (s, 3H, H12). ^13^C NMR (400 MHz, CDCl_3_): δ 151.4 (C14, C18), 150.6 (C16), 150.0 (C13), 137.9 (C24), 137.0 (C6), 136.4 (C2), 135.0 (C3), 130.0 (C21), 129.4 (C20 and C22), 129.3 (C4 and C8), 127.3 (C5 and C7), 126.9 (C19 and C23), 123.6 (C11 *of* imidazolyl), 122.4 (C15 and C17), 121.4 (C10 *of* imidazolyl), 114.6 (C1), 54.5 (C9), 38.2 (C12). Anal. Calcd for C_32_H_29_Cl_2_N_3_Pd: C, 60.73; H, 4.62; N, 6.64. Found: C, 60.52; H, 4.48; N, 6.52.
Geometry. All e.s.d.'s (except the e.s.d. in the dihedral angle between two l.s. planes) are estimated using the full covariance matrix. The cell e.s.d.'s are taken into account individually in the estimation of e.s.d.'s in distances, angles and torsion angles; correlations between e.s.d.'s in cell parameters are only used when they are defined by crystal symmetry. An approximate (isotropic) treatment of cell e.s.d.'s is used for estimating e.s.d.'s involving l.s. planes.

### Fractional atomic coordinates and isotropic or equivalent isotropic displacement parameters (Å^2^)

**Table:**

	*x*	*y*	*z*	*U*_iso_*/*U*_eq_	
Pd1	0.07483 (2)	0.20492 (2)	0.94817 (2)	0.02792 (4)	
Cl1	0.25183 (7)	0.15753 (5)	1.08527 (4)	0.05106 (13)	
Cl2	−0.11307 (6)	0.25396 (4)	0.81746 (3)	0.03823 (10)	
N3	0.17729 (19)	0.11549 (12)	0.83251 (11)	0.0321 (3)	
N2	0.0464 (2)	0.39712 (13)	1.10216 (12)	0.0370 (3)	
N1	−0.17080 (19)	0.25232 (13)	1.09351 (11)	0.0334 (3)	
C5	0.2103 (3)	0.47133 (16)	1.08373 (16)	0.0429 (4)	
H5A	0.1881	0.5452	1.0705	0.051*	
H5B	0.2460	0.4386	1.0177	0.051*	
C24	0.3205 (2)	−0.12292 (16)	0.51023 (13)	0.0363 (4)	
H24	0.2130	−0.1060	0.4829	0.044*	
C15	0.4285 (2)	0.07047 (14)	0.76901 (13)	0.0309 (3)	
H15	0.5536	0.0852	0.7759	0.037*	
C8	0.4998 (3)	0.45500 (17)	1.34428 (16)	0.0457 (5)	
H8	0.4936	0.4133	1.4038	0.055*	
C19	0.4004 (2)	−0.08203 (14)	0.61662 (13)	0.0305 (3)	
C3	−0.0648 (3)	0.42689 (18)	1.17048 (17)	0.0487 (5)	
H3	−0.0477	0.4979	1.2132	0.058*	
C20	0.5571 (2)	−0.10877 (15)	0.65431 (14)	0.0353 (4)	
H20	0.6125	−0.0824	0.7267	0.042*	
C21	0.6333 (3)	−0.17316 (16)	0.58797 (16)	0.0424 (4)	
H21	0.7408	−0.1905	0.6147	0.051*	
C12	0.7988 (3)	0.56108 (19)	1.43218 (19)	0.0500 (5)	
H12	0.7857	0.5128	1.4866	0.060*	
C17	0.1405 (2)	−0.02490 (17)	0.68094 (15)	0.0405 (4)	
H17	0.0623	−0.0784	0.6261	0.049*	
C23	0.3973 (3)	−0.18787 (16)	0.44476 (14)	0.0419 (4)	
H23	0.3415	−0.2158	0.3727	0.050*	
C2	−0.2001 (3)	0.33722 (19)	1.16486 (16)	0.0464 (5)	
H2	−0.2982	0.3322	1.2025	0.056*	
C14	0.3524 (2)	0.13006 (14)	0.83849 (13)	0.0313 (3)	
H14	0.4277	0.1845	0.8938	0.038*	
C4	−0.0199 (2)	0.28953 (14)	1.05559 (12)	0.0293 (3)	
C16	0.3223 (2)	−0.01184 (14)	0.68806 (13)	0.0307 (3)	
C9	0.6520 (2)	0.53798 (15)	1.34155 (15)	0.0381 (4)	
C18	0.0733 (2)	0.03931 (17)	0.75286 (15)	0.0414 (4)	
H18	−0.0511	0.0293	0.7458	0.050*	
C10	0.6572 (3)	0.59587 (16)	1.25243 (18)	0.0442 (5)	
H10	0.7605	0.6522	1.2475	0.053*	
C13	0.9444 (3)	0.6404 (2)	1.4456 (2)	0.0688 (7)	
H13A	0.9643	0.6910	1.3936	0.083*	
H13B	1.0309	0.6478	1.5074	0.083*	
C7	0.3571 (3)	0.43155 (17)	1.26257 (16)	0.0458 (5)	
H7	0.2548	0.3740	1.2666	0.055*	
C1	−0.2837 (2)	0.13842 (17)	1.06416 (16)	0.0420 (4)	
H1A	−0.2245	0.0847	1.0957	0.050*	
H1B	−0.3069	0.1198	0.9855	0.050*	
H1C	−0.3958	0.1340	1.0916	0.050*	
C11	0.5146 (3)	0.57258 (16)	1.17113 (17)	0.0420 (4)	
H11	0.5212	0.6133	1.1110	0.050*	
C6	0.3612 (2)	0.49074 (14)	1.17516 (15)	0.0364 (4)	
C22	0.5534 (3)	−0.21261 (16)	0.48248 (16)	0.0445 (5)	
H22	0.6062	−0.2564	0.4366	0.053*	

### Atomic displacement parameters (Å^2^)

**Table:**

	*U*^11^	*U*^22^	*U*^33^	*U*^12^	*U*^13^	*U*^23^
Pd1	0.02640 (7)	0.03243 (7)	0.02598 (7)	0.00963 (5)	0.00443 (4)	0.00293 (5)
Cl1	0.0572 (3)	0.0779 (4)	0.0306 (2)	0.0436 (3)	0.0032 (2)	0.0073 (2)
Cl2	0.0344 (2)	0.0448 (2)	0.0344 (2)	0.01076 (18)	−0.00241 (16)	0.00783 (17)
N3	0.0300 (7)	0.0359 (7)	0.0292 (7)	0.0078 (6)	0.0048 (5)	−0.0002 (6)
N2	0.0376 (8)	0.0355 (8)	0.0384 (8)	0.0100 (6)	0.0104 (6)	0.0001 (6)
N1	0.0301 (7)	0.0385 (8)	0.0347 (7)	0.0119 (6)	0.0097 (6)	0.0055 (6)
C5	0.0469 (11)	0.0357 (9)	0.0435 (10)	0.0038 (8)	0.0093 (8)	0.0057 (8)
C24	0.0365 (9)	0.0418 (9)	0.0276 (8)	0.0062 (7)	0.0030 (7)	0.0018 (7)
C15	0.0274 (8)	0.0335 (8)	0.0288 (7)	0.0026 (6)	0.0052 (6)	0.0003 (6)
C8	0.0509 (12)	0.0423 (10)	0.0433 (10)	0.0062 (9)	0.0134 (9)	0.0084 (8)
C19	0.0313 (8)	0.0314 (8)	0.0265 (7)	0.0036 (6)	0.0063 (6)	0.0014 (6)
C3	0.0529 (12)	0.0475 (11)	0.0493 (11)	0.0202 (10)	0.0173 (9)	−0.0060 (9)
C20	0.0399 (9)	0.0368 (9)	0.0283 (8)	0.0094 (7)	0.0039 (7)	0.0027 (7)
C21	0.0424 (10)	0.0402 (10)	0.0480 (10)	0.0160 (8)	0.0084 (8)	0.0062 (8)
C12	0.0462 (11)	0.0481 (11)	0.0577 (12)	0.0158 (9)	0.0108 (10)	0.0030 (10)
C17	0.0305 (9)	0.0487 (11)	0.0350 (9)	0.0044 (8)	0.0003 (7)	−0.0104 (8)
C23	0.0528 (12)	0.0415 (10)	0.0270 (8)	0.0041 (9)	0.0086 (8)	−0.0019 (7)
C2	0.0428 (11)	0.0583 (12)	0.0437 (10)	0.0205 (9)	0.0169 (9)	0.0014 (9)
C14	0.0297 (8)	0.0329 (8)	0.0283 (7)	0.0041 (6)	0.0032 (6)	0.0002 (6)
C4	0.0269 (7)	0.0341 (8)	0.0288 (7)	0.0111 (6)	0.0047 (6)	0.0045 (6)
C16	0.0307 (8)	0.0336 (8)	0.0260 (7)	0.0048 (6)	0.0051 (6)	0.0020 (6)
C9	0.0361 (9)	0.0329 (9)	0.0465 (10)	0.0110 (7)	0.0126 (8)	−0.0019 (8)
C18	0.0271 (8)	0.0521 (11)	0.0400 (9)	0.0069 (8)	0.0025 (7)	−0.0067 (8)
C10	0.0361 (10)	0.0341 (9)	0.0621 (12)	0.0044 (8)	0.0162 (9)	0.0051 (9)
C13	0.0519 (14)	0.0694 (16)	0.0778 (17)	0.0064 (12)	−0.0015 (13)	0.0109 (14)
C7	0.0427 (11)	0.0412 (10)	0.0475 (11)	−0.0039 (8)	0.0107 (9)	0.0073 (8)
C1	0.0322 (9)	0.0443 (10)	0.0498 (11)	0.0056 (8)	0.0108 (8)	0.0115 (8)
C11	0.0445 (11)	0.0327 (9)	0.0510 (11)	0.0075 (8)	0.0167 (9)	0.0100 (8)
C6	0.0396 (9)	0.0281 (8)	0.0423 (9)	0.0085 (7)	0.0133 (8)	0.0000 (7)
C22	0.0563 (12)	0.0360 (9)	0.0436 (10)	0.0128 (9)	0.0178 (9)	0.0002 (8)

### Geometric parameters (Å, º)

**Table:**

Pd1—Cl1	2.2901 (5)	C20—H20	0.9500
Pd1—Cl2	2.2957 (4)	C20—C21	1.381 (3)
Pd1—N3	2.0938 (14)	C21—H21	0.9500
Pd1—C4	1.9532 (16)	C21—C22	1.385 (3)
N3—C14	1.340 (2)	C12—H12	0.9500
N3—C18	1.342 (2)	C12—C9	1.474 (3)
N2—C5	1.456 (2)	C12—C13	1.299 (3)
N2—C3	1.387 (2)	C17—H17	0.9500
N2—C4	1.346 (2)	C17—C16	1.393 (2)
N1—C2	1.390 (2)	C17—C18	1.379 (3)
N1—C4	1.335 (2)	C23—H23	0.9500
N1—C1	1.455 (2)	C23—C22	1.374 (3)
C5—H5A	0.9900	C2—H2	0.9500
C5—H5B	0.9900	C14—H14	0.9500
C5—C6	1.505 (3)	C9—C10	1.389 (3)
C24—H24	0.9500	C18—H18	0.9500
C24—C19	1.398 (2)	C10—H10	0.9500
C24—C23	1.381 (3)	C10—C11	1.377 (3)
C15—H15	0.9500	C13—H13A	0.9500
C15—C14	1.370 (2)	C13—H13B	0.9500
C15—C16	1.396 (2)	C7—H7	0.9500
C8—H8	0.9500	C7—C6	1.379 (3)
C8—C9	1.386 (3)	C1—H1A	0.9800
C8—C7	1.380 (3)	C1—H1B	0.9800
C19—C20	1.389 (3)	C1—H1C	0.9800
C19—C16	1.473 (2)	C11—H11	0.9500
C3—H3	0.9500	C11—C6	1.389 (3)
C3—C2	1.330 (3)	C22—H22	0.9500
			
Cl1—Pd1—Cl2	176.789 (17)	C18—C17—C16	120.49 (15)
N3—Pd1—Cl1	91.21 (4)	C24—C23—H23	119.6
N3—Pd1—Cl2	91.74 (4)	C22—C23—C24	120.84 (17)
C4—Pd1—Cl1	89.00 (5)	C22—C23—H23	119.6
C4—Pd1—Cl2	88.05 (5)	N1—C2—H2	126.5
C4—Pd1—N3	179.52 (6)	C3—C2—N1	107.06 (17)
C14—N3—Pd1	120.15 (10)	C3—C2—H2	126.5
C14—N3—C18	117.38 (15)	N3—C14—C15	123.39 (14)
C18—N3—Pd1	122.42 (12)	N3—C14—H14	118.3
C3—N2—C5	124.98 (16)	C15—C14—H14	118.3
C4—N2—C5	125.18 (15)	N2—C4—Pd1	127.87 (12)
C4—N2—C3	109.84 (16)	N1—C4—Pd1	125.92 (12)
C2—N1—C1	126.16 (16)	N1—C4—N2	106.14 (14)
C4—N1—C2	109.98 (15)	C15—C16—C19	121.06 (15)
C4—N1—C1	123.85 (14)	C17—C16—C15	116.29 (15)
N2—C5—H5A	108.7	C17—C16—C19	122.64 (14)
N2—C5—H5B	108.7	C8—C9—C12	119.2 (2)
N2—C5—C6	114.40 (16)	C8—C9—C10	117.57 (19)
H5A—C5—H5B	107.6	C10—C9—C12	123.22 (18)
C6—C5—H5A	108.7	N3—C18—C17	122.41 (17)
C6—C5—H5B	108.7	N3—C18—H18	118.8
C19—C24—H24	119.9	C17—C18—H18	118.8
C23—C24—H24	119.9	C9—C10—H10	119.6
C23—C24—C19	120.21 (18)	C11—C10—C9	120.80 (18)
C14—C15—H15	120.0	C11—C10—H10	119.6
C14—C15—C16	119.97 (15)	C12—C13—H13A	120.0
C16—C15—H15	120.0	C12—C13—H13B	120.0
C9—C8—H8	119.2	H13A—C13—H13B	120.0
C7—C8—H8	119.2	C8—C7—H7	119.6
C7—C8—C9	121.6 (2)	C6—C7—C8	120.78 (18)
C24—C19—C16	121.42 (16)	C6—C7—H7	119.6
C20—C19—C24	118.45 (16)	N1—C1—H1A	109.5
C20—C19—C16	120.13 (14)	N1—C1—H1B	109.5
N2—C3—H3	126.5	N1—C1—H1C	109.5
C2—C3—N2	106.98 (17)	H1A—C1—H1B	109.5
C2—C3—H3	126.5	H1A—C1—H1C	109.5
C19—C20—H20	119.6	H1B—C1—H1C	109.5
C21—C20—C19	120.86 (16)	C10—C11—H11	119.3
C21—C20—H20	119.6	C10—C11—C6	121.41 (19)
C20—C21—H21	119.9	C6—C11—H11	119.3
C20—C21—C22	120.15 (19)	C7—C6—C5	123.98 (17)
C22—C21—H21	119.9	C7—C6—C11	117.86 (19)
C9—C12—H12	116.5	C11—C6—C5	118.16 (18)
C13—C12—H12	116.5	C21—C22—H22	120.3
C13—C12—C9	126.9 (2)	C23—C22—C21	119.49 (18)
C16—C17—H17	119.8	C23—C22—H22	120.3
C18—C17—H17	119.8		
			
Pd1—N3—C14—C15	176.64 (14)	C2—N1—C4—Pd1	−177.10 (14)
Pd1—N3—C18—C17	−175.65 (16)	C2—N1—C4—N2	−0.1 (2)
N2—C5—C6—C7	9.5 (3)	C14—N3—C18—C17	1.9 (3)
N2—C5—C6—C11	−171.03 (16)	C14—C15—C16—C19	−176.13 (16)
N2—C3—C2—N1	0.4 (2)	C14—C15—C16—C17	2.4 (3)
C5—N2—C3—C2	179.58 (19)	C4—N2—C5—C6	−105.5 (2)
C5—N2—C4—Pd1	−2.7 (3)	C4—N2—C3—C2	−0.4 (2)
C5—N2—C4—N1	−179.71 (17)	C4—N1—C2—C3	−0.2 (2)
C24—C19—C20—C21	0.7 (3)	C16—C15—C14—N3	−1.3 (3)
C24—C19—C16—C15	−149.10 (17)	C16—C19—C20—C21	−179.11 (16)
C24—C19—C16—C17	32.4 (3)	C16—C17—C18—N3	−0.6 (3)
C24—C23—C22—C21	0.9 (3)	C9—C8—C7—C6	−0.3 (3)
C8—C9—C10—C11	1.2 (3)	C9—C10—C11—C6	−0.1 (3)
C8—C7—C6—C5	−179.13 (19)	C18—N3—C14—C15	−1.0 (3)
C8—C7—C6—C11	1.4 (3)	C18—C17—C16—C15	−1.6 (3)
C19—C24—C23—C22	−0.5 (3)	C18—C17—C16—C19	176.99 (19)
C19—C20—C21—C22	−0.3 (3)	C10—C11—C6—C5	179.26 (18)
C3—N2—C5—C6	74.5 (3)	C10—C11—C6—C7	−1.2 (3)
C3—N2—C4—Pd1	177.26 (14)	C13—C12—C9—C8	−175.0 (2)
C3—N2—C4—N1	0.3 (2)	C13—C12—C9—C10	4.7 (3)
C20—C19—C16—C15	30.7 (2)	C7—C8—C9—C12	178.69 (19)
C20—C19—C16—C17	−147.77 (19)	C7—C8—C9—C10	−1.0 (3)
C20—C21—C22—C23	−0.5 (3)	C1—N1—C2—C3	178.86 (19)
C12—C9—C10—C11	−178.52 (18)	C1—N1—C4—Pd1	3.8 (2)
C23—C24—C19—C20	−0.3 (3)	C1—N1—C4—N2	−179.15 (16)
C23—C24—C19—C16	179.52 (16)		

### Hydrogen-bond geometry (Å, º)

**Table:**

*D*—H···*A*	*D*—H	H···*A*	*D*···*A*	*D*—H···*A*
C20—H20···Cl1^i^	0.95	2.81	3.6021 (18)	142
C23—H23···Cl2^ii^	0.95	2.74	3.6537 (19)	162

Symmetry codes: (i) −*x*+1, −*y*, −*z*+2; (ii) −*x*, −*y*, −*z*+1.
